# Advancement in Determination of Phthalate Metabolites by Gas Chromatography Eliminating Derivatization Step

**DOI:** 10.3389/fchem.2019.00928

**Published:** 2020-01-15

**Authors:** Maciej Tankiewicz, Ewa Olkowska, Andrzej Berg, Lidia Wolska

**Affiliations:** Department of Environmental Toxicology, Faculty of Health Sciences With Institute of Maritime and Tropical Medicine, Medical University of Gdansk, Gdansk, Poland

**Keywords:** phthalate metabolites, thermal stability, injection conditions, gas chromatography, separation, analytical method, derivatization

## Abstract

A gas chromatography-mass spectrometry (GC–MS) method to determine polar and thermally unstable phthalate metabolites [monomethyl phthalate–MMP, monoethyl phthalate–MEP, mono-*n*-butyl phthalate–MnBP, mono-(2-ethylhexyl) phthalate–MEHP] has been developed. This is the first report presenting the separation of monophthalates without derivatization step and any additional equipment or special injection port. Injection parameters (temperature, pressure, time, and volume of injection), chromatographic separation (retention gap, temperature program), and MS detection/identification (working parameters, ion selection) were investigated. Mechanisms and phenomena occurring under different conditions in the GC injector were evaluated and discussed. The limits of detection (LODs) of MMP, MEP, MnBP, MEHP in the protocol were 0.049, 0.036, 0.038, and 0.029 ng (*per* 2 μL of injection), respectively. The response of the monophthalates was found to be linear in the tested concentration range (for MMP: 0.15–100 ng, MEP and MnBP: 0.11–100 ng, MEHP: 0.087–100 ng *per* 2 μL) with the coefficient of determination higher than 0.9817 and inter-day precision in the range of 1.4–5.4%. The developed method is fast, easy and repeatable. Moreover, it allows for the elimination of derivatization agents, reduction of toxic waste production and simplification of analytical procedure.

## Introduction

Phthalate diesters, generally known as phthalates, have been intensively used since the 1960s. Their chemical structure consists of di-alkyl ester of 1,2-benzenedicarboxylic acid. Depending on the length and isomeric structure of the hydrocarbon chain, they exhibit different characteristics (Calafat and McKee, 2006). Compounds with low molecular weight (dimethyl phthalate DMP, diethyl phthalate DEP, dibutyl phthalate DBP) possess emulsifying and softening properties. Higher alkyl phthalates [di-(2-ethylhexyl) phthalate DEHP and di-isononyl phthalate DiNP] are principally used in polymer production (especially in polyvinyl chloride–PVC) as plasticizers (Chang-Liao et al., 2013; Dewalque et al., 2014). They are widely used in many personal care and consumer products, such as cosmetics, lubricants, perfumes, and lotions (Silva et al., 2005; Mankidy et al., 2013). Moreover, phthalates have found a variety of applications in pharmaceutical products, construction materials, paints, flooring, and wall coverings, food packaging materials, electronics, and medical devices. Therefore, contact with these substances is ubiquitous and practically continuous in different spheres of life, causing extreme risk of exposure (Jepsen et al., 2004; Oca et al., 2016; Koch et al., 2017; Li and Suh, 2019; Heffernan et al., 2020).

At room temperature, phthalates are oily liquids with low volatility and varying miscibility with polymers. They contain polar groups in their structure and are characterized by high solubility. Their addition reduces intermolecular interactions and increases the mobility of polymer chains (Przybylinska and Wyszkowski, 2016). Phthalates do not form covalent bonds with the polymers they are mixed with. Therefore, they can freely migrate to the surface of products and further into food and beverages in contact with these surfaces. Consequently, this has led to their widespread dispersion into the environment, providing an easy source of human exposure by inhalation, ingestion, dermal absorption, or even intravenous route (Hogberg et al., 2008; Orecchio et al., 2014; Bao et al., 2015; Giovanoulis et al., 2018; Li and Suh, 2019).

On exposure in humans, phthalate diesters are quickly hydrolyzed to monoester forms, which consist of one free reactive functional carboxylic acid and one ester group. In the case of low molecular weight compounds (for example, diethyl phthalate), the metabolism ends with the hydrolytic monoester form. However, for higher molecular weight phthalates [for example, di-(2-ethylhexyl) phthalate, di-isononyl phthalate], metabolism continues with transformation to oxidative products (oxidation of alkyl chain), which are more hydrophilic. Monoesters and oxidative metabolites may directly be excreted into urine or conjugate with α-D-glucuronic acid (phase II biotransformation reactions) thus raising water solubility and therefore increased urinary excretion (Nuti et al., 2005; Calafat and McKee, 2006; Mose et al., 2007; Hogberg et al., 2008; Kondo et al., 2010). Studies on human phthalate exposure and risk assessment require firstly an estimation of phthalate metabolite concentrations in tested samples. And for that, appropriate analytical procedures are needed. The determination of monoester metabolites instead of diester compounds as biomarkers aims to compare relative exposures to various phthalates in epidemiological studies (Frederiksen et al., 2007; Mose et al., 2007; Tranfo et al., 2012). Moreover, it can help overcome the contamination problems from laboratory equipment releasing diesters, such as chemical and solvent packaging, gloves, rubber caps and seals, syringes, pipette tips, filters, stir bars, and vials. Even the chromatographic system (for example injector septa, ferrules, vial caps, washing solvent, syringe, and injector liner) and laboratory air may be sources of contamination (Reid et al., 2007; Oca et al., 2016). Another advantage of utilizing the hydrolytic phthalate monoesters as biomarkers is that they are generally considered as biologically active molecules (Hauser and Calafat, 2005). Zhang et al. proposed lactic acid as another biomarker of phthalates exposure, which was extracted from cells exposed to mono-(2-ethylhexyl) phthalate. The determination of this biomarker (potential energy source for cancer cells) could be useful in studies on mitochondrial function and glycolytic conditions in cells (Zhang et al., 2018).

In literature, many analytical methodologies for the determination of monoester phthalates (MP) in various matrices can be found, such as biological (serum, urine, saliva, seminal plasma, breast milk, sperm, etc.), PCV products, environmental, packaging materials, plastic toys, food, and beverages (Mose et al., 2007; Guo et al., 2010; Bamai et al., 2015; Nassan et al., 2017; Del Bubba et al., 2018). Most of them include extraction, cleanup and quantification by using chromatographic methods. The techniques used for isolation and/or enrichment from solid and liquid samples include extraction with organic solvents, solid-phase extraction, solid-phase microextraction, and other modifications of solvent microextraction. For qualitative and quantitative analysis, chromatography coupled with mass spectrometry (MS) is used. Other detectors can be also used. However, analytical methods, which do not include MS detection, are generally less selective and sensitive (Yang et al., 2015; Kumar and Sivaperumal, 2016; González-Sálamo et al., 2018). High performance liquid chromatography (HPLC) is the most common technique. Analytical procedures using isotope dilution HPLC coupled with tandem mass spectrometry (MS/MS) for detecting trace levels of phthalate metabolites in biological samples have been mostly reported (Silva et al., 2005; Chang-Liao et al., 2013; Bernard et al., 2014; Dewalque et al., 2014; Yang et al., 2015; Kumar and Sivaperumal, 2016; González-Sálamo et al., 2018). Nowadays, isotope dilution mass spectrometry is considered as the most appropriate and precise method for analysis at very low concentrations, corresponding to metabolite levels in real samples. However, such analyzes involve very high costs of deuterated standard solutions and equipment maintenance (Frederiksen et al., 2007; Yang et al., 2015). The problematic issue with triple quad MS is the ion suppression effect, related to interferences from sample matrix, coeluting compounds, and cross-talk. These molecules can affect the efficiency of droplet formation or droplet evaporation, which consequently influences on the amount of charged ion in the gas phase that finally reaches to the detector. As a result, deterioration of analytical methodology parameters (such as limit of detection, precision and accuracy) can be observed (Majumdar, 2005). In contrast, electron ionization (EI), the most commonly used gas chromatography (GC-MS) ionization technique, which is very powerful, reproducible and does not suffer from ion suppression effect, can be a good alternative for this purpose (Niino et al., 2002; Guo et al., 2010; Rastkari and Ahmadkhaniha, 2013). However, when using GC, according to literature, the derivatization step is mandatory to convert phthalates to volatile derivatives, which is based on the methylation (-COOCH_3_) or silylation (-COOSiR_3_) process of carboxylic acid group. Without blocking the highly polar acid group, analytes can adsorb during GC analysis. Moreover, commonly used derivatizing agents are highly toxic and potentially explosive. In addition, their use implies an additional step in the analytical procedure, which can be a source of complications and errors (Niino et al., 2002; Chen et al., 2012; Kumar and Sivaperumal, 2016). For example, use of derivatizing agents with acidic nature (e.g., fluorinated anhydrides) requires removal of excess agent or byproducts before GC analysis to prevent the deterioration of the chromatographic column (Orata, 2012). In addition, use of some silylation agents and the related silylated byproducts may deteriorate stationary phases (e.g., polyethylene glycol-type columns) and analytes separation cannot be done on such columns (Moldoveanu and David, 2019). It should be noticed, that the silyl derivatization technique is not selective and allows determining the sum of mono-alkyl phthalate esters, phthalic acid, and phthalic acid esters in tested samples. Thus, to determine the real level of monophthalates additional measurements have to be performed (Net et al., 2015). The aim of this study was the development of a new methodology for quantitative and qualitative analysis of the most common phthalate monoesters by using GC-MS. The analytical procedure was optimized and validated to obtain the highest efficiency in terms of linearity, limits of detection (LODs), limits of quantification (LOQs), and inter-day precision. This is the first report presenting the applicability of a GC technique for the immediate determination of phthalate metabolites without a derivatization step and use of additional equipment or special injection port.

## Materials and Methods

### Reagents and Standard Solutions

The following chemicals were used as analytical standards: monomethyl phthalate (MMP), monoethyl phthalate (MEP), mono-*n*-butyl phthalate (MnBP), and mono-(2-ethylhexyl) phthalate (MEHP), which were purchased in solid form from AccuStandard, Inc. (USA). Chemical structures and physicochemical properties of determined monophthalates (MP) are presented in Supplementary Material (see Table S1 and Section 1.1). The collected data about monophthalates were used to develop a suitable GC-MS method for their determination. Methanol (GC purity 99.8%) from Merck KGaA (Darmstadt, Germany) was used. Each solid standard was dissolved in 2 mL of methanol at the 50 mg/mL concentration level.

Working standard solutions for calibration were prepared by dilution of standard stock solutions with methanol to obtain following concentrations per 2 μL of injection: 0.05, 0.1, 0.8, 1.2 (MEHP), 2 (MEHP), 4 (MnBP, MEHP), 10 (MnBP, MEHP), 20, 30, 50, and 100 ng. All standard solutions were stored in freezer at 4°C. Helium with 99.999995% purity (Air Products, Poland) was used as carrier gas for the chromatographic analysis. To eliminate contamination of glassware and other laboratory equipment from phthalates, all used vessels were washed twice with acetone and hexane, and then baked in oven at 150°C for 1 h. In the course of analytical proceedings, attempts were made to eliminate plastic products.

### Instrumentation and Data Analysis

Determination of monophthalates was carried out by gas chromatography (GC-2010 PLUS, Shimadzu Corp., Japan) coupled with an autosampler (Auto Injector AOC−20ia, Shimadzu Corp., Japan) and mass spectrometer (MS-TQ8040, Shimadzu Corp., Japan). The injector was fitted with a single taper liner with 3.4 mm of internal diameter (ID) and 95 mm of length (Phenomenex, USA). Silane treated glass wool was obtained from Alltech Associates (Deerfield, USA). The gas chromatograph was equipped with DB-5 MS (phenyl arylene polymer virtually equivalent to a 5%-phenyl-methylpolysiloxane) capillary column (30 m length × 0.25 mm ID × 0.25 μm film thickness, Agilent Technologies, USA), connected with the retention gap. As a retention gap, the following 0.60 m of capillary columns were tested: uncoated deactivated fused silica tubing (0.25 mm ID, Zebron Guard Column Kit, Phenomenex, USA), Rtx®-200 (trifluoropropylmethyl polysiloxane, 0.25 mm ID × 1.0 μm of film thickness, Restek Corporation, USA) and SUPELCOWAX™-10 (polyethylene glycol, 0.25 mm ID × 1.0 μm of film thickness, Supelco, USA). The mass spectrometer in EI mode was operated with 70 V of ionization voltage and 150 μA of emission current. Helium carrier gas at constant flow rate of 1.0 mL/min was used. Control and operation of the chromatographic system was performed using *GCMS Real Time Analysis* software (Shimadzu Corp., Japan). GC-MS data were processed with the *GCMS Postrun Analysis* software (Shimadzu Corp., Japan). The confirmation of compound identifications was performed by using similarity searches in the National Institute of Standards and Technology MS database (NIST 11).

During studies the following analysis parameters were investigated: condition of injection (temperature, pressure, volume of injection), chromatographic separation (temperature program) and mass spectrometry detection/identification (working parameters, ion selection). The conditions of analytical protocol were evaluated and programmed based on experimental results. Statistical analysis of obtained results was performed using Microsoft Excel™ 2010 (USA).

### Analytical Performance

In order to determine monophthalates with the proposed analytical protocol, selected validation parameters were evaluated: linearity range, LOD, LOQ, and inter-day precision. Linear regression analysis by the least-squares method of peak area ratios corresponding to proper analytes against different concentration levels was used to check analyte responses in the range of proposed method applications. For LOD and LOQ estimation, external calibration curves were prepared for each monophthalate (three replicates of each) using SIM mode. LODs and LOQs were calculated based on the residual standard deviation of the calibration function (Sa) and the slope of the calibration curve (b) at low concentrations according to the formulas LOD = 3.3 ^*^ (Sa/b) and LOQ = 10 ^*^ (Sa/b), respectively. Inter-day precision (repeatability) was evaluated by analysis of standard solutions at four different levels (with five replicates). The repeatability was described by the coefficient of variability (% CV) (Analytical Methods Committee, 1994; Taverniers et al., 2004; ICH Harmonised Tripartite Guideline, 2005).

## Results and Discussion

### Evaluation of Injection Conditions

**Figure 1:**
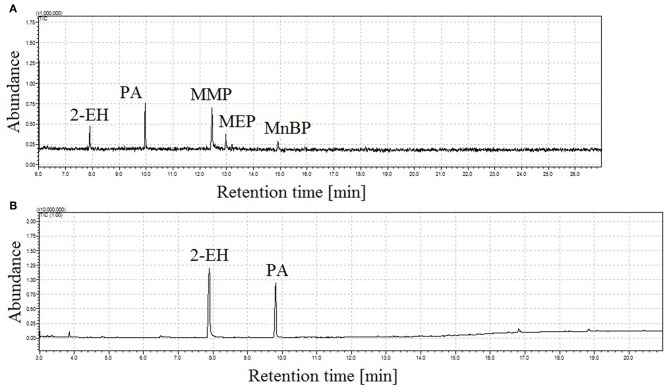
Total ion chromatograms of a four monophthalates mixture (100 ng in 2 μL of injection) obtained under classical GC injector conditions, splitless working mode of injector, injection time = 1 min, injection temp.: 190°C **(A)** and 250°C **(B)**; peak with retention time *t*_r_ = 7.9 min corresponds to 2-ethylhexanol (2-EH), *t*_r_ = 9.8 min to phthalic anhydride (PA), *t*_r_ = 12.4 min monomethyl phthalate (MMP), *t*_r_ = 13.0 min monoethyl phthalate (MEP), and *t*_r_ = 14.9 min mono-*n*-butyl phthalate (MnBP).

Using classical injector working conditions, in splitless mode, monoesters degrade to phthalic anhydride (PA) and 2-ethylhexanol (2-EH), which was formed during degradation of MEHP. No peaks for other groups from degraded monophthalates were visible in the chromatogram due to the solvent cut time applied. In addition, mass spectral data from *m/z* 45 were collected. In Figure 1, chromatograms obtained with classical splitless injection at two different temperatures are presented. At 190°C (Figure 1A) not all peaks corresponding to analytes were observed and degradation products from monophthalates were also detected. Simultaneously, the percentage of molecules of decomposed monophthalates increases with the temperature of the injector, as seen in Figure 1B.

The solution introduced by micro-syringe into the injector uses a polar solvent (methanol), which due to weak electrostatic interactions, forms a solvation shell around polar phthalic acid monoesters. The monophthalate molecules are energetically stabilized by the solvation shell. Therefore, there are no ions on the surface of the solution, only pure solvent. After injection of the monophthalates solution into a hot injector (about 250°C), in which the carrier gas flows at a rate ~1 mL/min and pressure usually does not exceed 62 kPa, the evaporation of the solution components into the gas phase takes place. The first compound to evaporate and at the largest quantities (according to the Gibbs–Konowalow law) is methanol (boiling point = 65°C). Rapidly evaporating methanol (~2 μL) causes a temporary pressure increase in the injector and a local temperature decrease of the carrier gas stream (Grob and Barry, 2004; Atkins and De Paula, 2014).

A change in pressure and temperature conditions reduces the propensity of methanol molecules entering the gas phase, since they are involved in solvation of monophthalates. However, some of the solvent molecules evaporate which destabilizes the monophthalates molecules and leads to their degradation. Increasing the pressure in the injection port increases the flow rate of the carrier gas (around 2.8 mL/min) and the boiling point of the mixture (according to Clapeyron's law), and at the same time reducing the evaporation rate of methanol. Thus, there is no thermal degradation of compounds. According to Boyle-Mariotte's law: the higher the pressure, the more densely packed the particles are and the volume of the gas phase (molar) decreases (Grob and Barry, 2004; Atkins and De Paula, 2014). Through these conditions, the solvated form of the mixture is stabilized. In addition, the lower injector temperature ensures efficient evaporation of the sample and simultaneously the provided heat does not cause thermal degradation of the compounds.

### Selection of the Proper Injection Conditions

A sample volume of 2 μL was injected in splitless mode into a deactivated glass liner (without glass wool). Single taper liner was used to obtain minimal sample dilution^1^. During experiments injections without and with glass wool in the liner were also tested. Glass wool may be used to improve the sample vaporization process by making it more gentle, but on the other hand it is an adsorptive material and particularly problematic for trace analyses with splitless injection^2^. However, the addition of glass wool in the liner did not improve the obtained results and further analysis were performed without it. Under standard conditions of splitless injection at 250°C degradation of analytes was observed. The peaks for phthalic anhydride and 2-ethylhexanol were present, which confirms thermal degradation of monophthalates. During experiments, lower injection temperatures were also tested, but the detector responses were too low for proper identification and obtained chromatographic peaks were splitted.

Splitless injection mode is associated with creation of a large solvent vapor cloud, which can escape from the liner or perform extraction of impurities from septa and *O*-rings. When more than 2 μL of sample volume was injected, higher sample losses were observed. This can be explained by the vapor volume being larger than the inlet liner volume, with extra vapor escaping. Moreover, analytes could adsorb on heated inlet components. Therefore, 2 μL of sample volume was selected for subsequent studies (Tienpont, 2004; Grob, 2007; Biedermann, 2014). However, injection of 2 μL of methanol at 250°C and under standard pressure (53 kPa) gives an expansion volume of 1,340 μL, which is significantly higher than the liner volume (862 μL). Calculations of expansion volumes under stationary conditions were performed based on the equation available on-line on the Agilent Technologies website in the document: Vapor Volume Calculations of Common Liquids and Solvents^3^. Above all, it should be noted that the injected sample comprises not only the pure solvent, but also the analytes. Moreover, processes undergoing in the injector have a dynamic character due to carrier gas flow.

**Figure 2:**
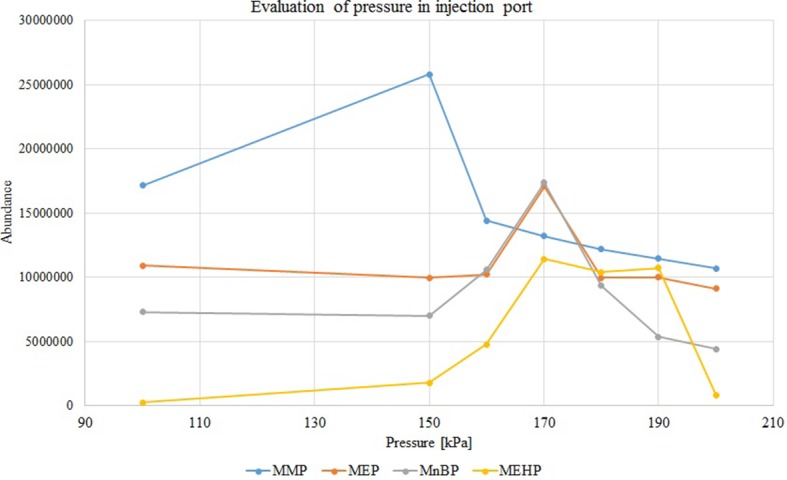
Variations in peak areas of monophthalates with increasing injection pressure (in splitless mode), concentration of each analyte 50 mg/L. MMP, monomethyl phthalate; MEP, monoethyl phthalate; MnBP, mono-*n*-butyl phthalate; MEHP, mono-(2-ethylhexyl) phthalate.

Further investigation was focused on the effects of pressure and temperature conditions on the chromatogram peak pattern (peak shape and area). According to those aspects, the selection of proper temperature and pressure could be the solution to ensure the vaporized sample remains inside the liner before transfer to the head of the analytical column. To compare the influence of pressure on the injection process, samples were injected at 50 kPa, 100 kPa and in the range from 150 to 200 kPa (with 10 kPa step increases). Higher-pressure injection (HPI) mode at 170 kPa allows for proper samples injection without derivatization stage (especially the heavier MEHP) and obtaining better chromatogram peak pattern. The effect of pressure change in the injection port is presented in the Figure 2.

**Figure 3:**
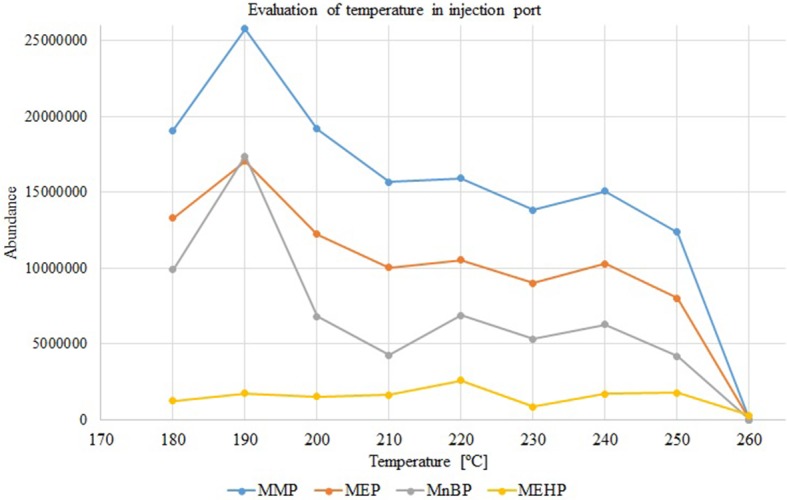
Variations in peak areas of monophthalates with increasing injection temperature (splitless and higher-pressure mode, *p* = 170 kPa), concentration of each analyte 50 mg/L. MMP, monomethyl phthalate; MEP, monoethyl phthalate; MnBP, mono-*n*-butyl phthalate; MEHP, mono-(2-ethylhexyl) phthalate.

During analysis in HPI mode, the influence of temperature on the separation process was also investigated (from 150 to 260°C with 10°C intervals). The increase in monophthalates peak area was observed with increasing injection temperature up to 190°C and gradually decreasing in the 200–260°C range (Figure 3). The thermal degradation of monophthalates was confirmed by the occurrence of phthalic anhydride and 2-ethylhexanol peaks. For this study, an injector temperature of 190°C was selected. Lower temperatures in the injection port caused splitting of chromatographic peaks, which confirms that provided energy was insufficient for effective evaporation of analytes. Moreover, higher-pressure injection mode at 170 kPa and 190°C allows for proper samples injection because formed vapor volume was 701.41 μL, which is slightly lower than liner volume (862 μL).

Additionally, splitless time was experimentally determined in the tested range from 30 s to 2 min (with 30 s steps). A splitless period of 1 min ensures the best injection conditions for samples due to the higher carrier gas flow (around 2.8 mL/min) instead of 1 mL/min in classical injection conditions.

### Application of Retention Gap

**Figure 4:**
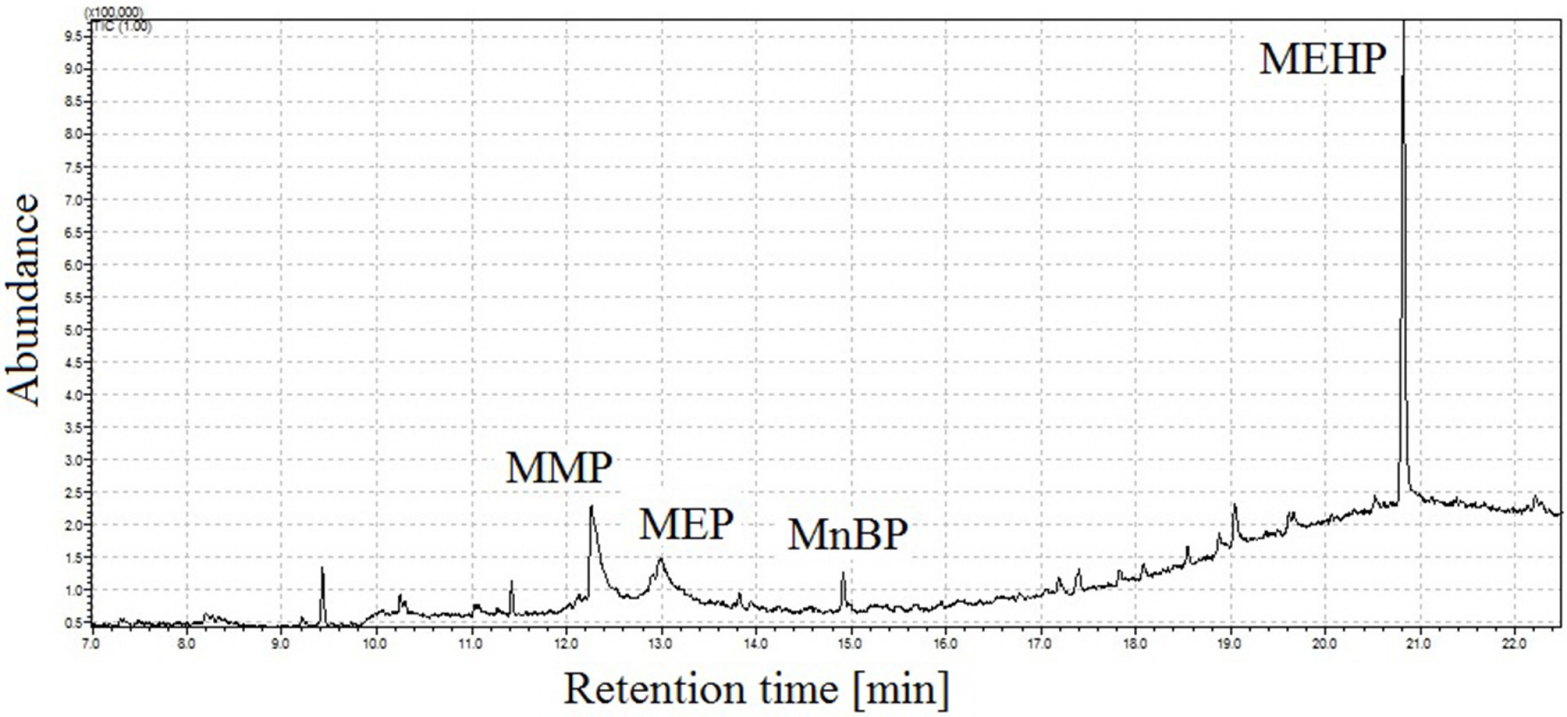
Total ion chromatogram of mixture of four monophthalates obtained under proposed conditions of GC injector, and, using uncoated fused silica between injector and chromatographic column. MMP, monomethyl phthalate; MEP, monoethyl phthalate; MnBP, mono-*n*-butyl phthalate; MEHP, mono-(2-ethylhexyl) phthalate.

To improve analytes peak shapes under optimized injection conditions (pressure: 170 kPa, temperature: 190°C) the introduction of a retention gap between injector and chromatographic column was tested. During the conducted trials, the retention gap was connected with the analytical column via glass connector. First experiments included use of uncoated deactivated fused silica tubing (maximum program temperature 340°C), which was coupled to the analytical column. Use of uncoated fused silica precolumn ensures analytes separation without their degradation, but obtained peak shapes were not satisfactory (Figure 4).

To improve peak shapes, a coated retention gap was introduced between the injector and capillary column. Two polar columns were tested: SUPELCOWAX™-10 and Rtx®-200 with maximum program temperature 280 and 310°C, respectively. With SUPELCOWAX™-10 column, a problem with separation of MEHP was observed due to the thermal limitation of the column (280°C). When using columns with different stationary phases in the same oven the isothermal limit has to be set to the lower of the two columns^4^.

After changing to Rtx®-200, the best peak shapes for all analytes were obtained. Moreover, an important aspect of using solvent focusing is the solvent wetting ability of the stationary phase in the analytical column. As described in the experiments, midpolar retention gap (Rtx®-200) with thick film was applied to improve focusing of polar solvent on the head, since the previously used methanol will not form an efficient zone of recondensed solvent in the analytical column. Introduction of the above mentioned retention gap eliminates differences of polarity between column stationary phase and the solvent, which can cause band broadening, peak splitting or poor resolution of analytes. Another reason to apply a polar retention gap was the non-polar character of the analytical column. Therefore, a non-polar solvent was not needed during GC analysis of monophthaletes.

Furthermore, the higher-pressure injection mode causes the transfer of the vaporized sample into the column, which prevents thermal degradation of monophthalates (Grob, 2007). As mentioned previously, higher pressure in the injector results in higher pressure differences between the column and injector, which causes higher transfer rate of vaporized sample to the head of the column. The presented experimental mechanism for the splitless injection process is summed in Supplementary Section 1.2.

### Selection of the Separation Conditions

Low initial GC oven temperatures (30, 40, and 50°C) were used to obtain the condensation of the solvent and film formation on the column surface, what results in easier release of analytes from the stationary phase (Poole, 2012; Biedermann, 2014). An initial GC oven temperature of 50°C was selected since it gives acceptable separation. This value fulfills the recommendation about selecting an initial oven temperature of 10–20°C below the boiling point of the solvent (methanol T_bp_ = 65°C) to achieve a fast condensation of the vapor (Poole, 2012; Biedermann, 2014). After holding the GC oven at low temperature for 1 and 2 min no improvement of analyte separation was observed.

Four different temperature rise values (7, 8, 10, 12°C/min) were tested and compared. Faster temperature program results in sharper peaks and shorter retention times of analytes without losses in their resolution and separation. The best chromatogram peak patterns (higher and sharper peaks) were obtained with an increase of 12°C/min and it allows to separation of MP and DP with symmetric peaks. As the final GC oven temperature, 280°C was selected to prevent degradation of analytes and stationary phase of the column.

### Selection of the MS Detection and Identification Conditions

After selecting the injection and GC separation parameters, the MS detection conditions have been tested and selected. The MS interface and ion source temperatures were maintained at 200 and 250°C, respectively. Full scan mode (SCAN, mass scan range 45–450 *m/z*) was used to estimate the time windows and target ions for the selected ion monitoring (SIM) mode for each monophthalate. For quantitation analysis of compounds, the SIM mode was used.

Total analysis time was estimated 23.17 min with 4 min of solvent cut time (to prevent saturation of the MS ion multiplier). For all monophthalates the most abundant ion was *m/z* 149, which originates from the protonated phthalic anhydride [C_8_H_4_O_3_H]^+^. This ion is similar to the one observed for diphthalates (Tienpont, 2004; Jeilani et al., 2011). At least three specific ions were selected for each analyte and used to identify the compound. The first ion, the most intensive, was used for measurement and the other two for confirmation (Table 1). One of the monitored ions for each MP corresponded to its molecular mass. Due to the problem with the ubiquity of phthalates and similarity of *m/z* ions, molecular masses corresponding to diphthalates were also monitored to be sure to identify proper peaks during integration and to eliminate possible errors.

**Table 1:** Parameters of MS detection for studied monophthalates.

**Analyte**	**Time windows [min]**	**Dwell time [ms]**	**Monitored ions [*****m/z*****]**	
			**Quantification**	**Confirmation**
MMP	7 ÷ 13.90	30	149	104, 180
MEP		30	149	176, 194, 222^*^
MnBP	13.90 ÷ 17.50	30	149	167, 65, 222, 278^*^
MEHP	17.50 ÷ 23.00	30	149	167, 70, 279, 390^*^

* *Ions corresponding to molecular mass of di-phthalates, MMP, monomethyl phthalate; MEP, monoethyl phthalate; MnBP, mono-n-butyl phthalate; MEHP, mono-(2-ethylhexyl) phthalate*.

**Figure 5:**
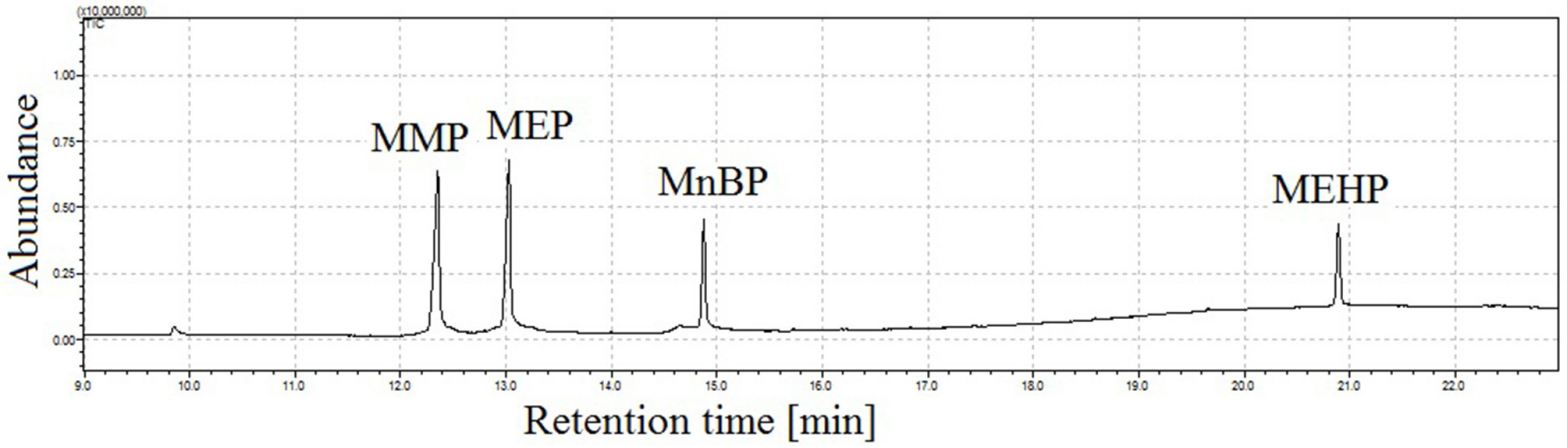
Total ion chromatogram of mixture of four monophthalates (100 ng in 2 μL of injection) obtained under proposed conditions of GC injector, splitless working mode of injector, injection time = 1 min, injection temp. = 190°C, injection pressure = 170 kPa; peak with retention time *t*_r_ = 12.4 min corresponds to monomethyl phthalate (MMP), *t*_r_ = 13.0 min monoethyl phthalate (MEP), *t*_r_ = 14.9 min mono-*n*-butyl phthalate (MnBP), and *t*_r_ = 20.9 min mono-(2-ethylhexyl) phthalate (MEHP).

Figure 5 illustrates the total ion chromatogram of a mixture of four monophthalates obtained by the proposed GC-MS method. During the experiments, a linear dependence of retention times of monophthalates on their boiling points was observed (see Figure S1). In addition, the elution of monoesters was noticed before the elution of corresponded di-phthalates.

### Validation Parameters of Developed Procedure

The method was validated by investigating the above-mentioned parameters. The basic validation parameters with the appropriate retention times of analytes are reported in Table 2. The data is reported in mass units *per* 2 μL of injection. The responses for MEHP (0.087–100 ng *per* 2 μL) and MMP, MEP, MnBP were found to be linear in the first and second concentration ranges, respectively (see Table 2). The calibration curves showed acceptable linearity with a coefficient of determination in the range of 0.9817–0.9993. The LOD and LOQ values for monophtalates ranged from 0.029 to 0.049 and from 0.087 to 0.15 ng, respectively. The developed method was found to be accurate as inter-day precision tests were between 1.4 and 5.4%.

**Table 2:** Basic validation data obtained for each phthalate metabolite by using GC-MS method.

**Analyte**	**Retention time [min]**	**Equation**	**R^**2**^**	**LOD [ng]**	**LOQ [ng]**	**CV [%] at LOQ level**	**Linearity range [ng]**	**CV [%]**
MMP	12.185	y = 9574.5 x – 1982.4	0.9993	0.049	0.15	5.4	0.15–20	7.3
		y = 81,562 x – 2·10^6^	0.9984				20–100	
MEP	13.079	y = 3248.6 x + 1,080	0.9817	0.036	0.11	3.8	0.11–20	6.2
		y = 73,544 x – 2·10^6^	0.9962				20–100	
MBP	14.680	y = 32,329 x – 23526	0.9984	0.038	0.11	2.8	0.11–10	9.1
		y = 36,362 x + 1368.3	0.9985				10–100	
MEHP	20.613	y = 32,329 x – 23,526	0.9984	0.029	0.087	1.4	0.087–100	5.1

MMP, monomethyl phthalate; MEP, monoethyl phthalate; MnBP, mono-n-butyl phthalate; MEHP, mono-(2-ethylhexyl) phthalate;

*R^2^, coefficient of determination; LOD, limit of detection; LOQ, limit of quantification; CV, coefficient of variability*.

The developed method concerns the final determination step in the course of analytical proceedings, which includes qualitative and quantitative analysis of monophthalates. In the case of biological samples or other kind of matrix, an isolation and/or enrichment step is necessary. Clean up of the obtained extract is also commonly applied. The proposed procedure is well-suited for this purpose. The most commonly used technique for the determination of thermally labile and polar organic compounds involves liquid chromatography, because their derivatization is avoided. Several literature examples of procedures using LC and GC techniques together with their basic validation data are presented in Table 3.

**Table 3:** Literature examples of analytical methodologies with their basic validation data used for the determination of monophthalates by LC and GC techniques.

**Analytes**	**Matrix**	**Sample preparation technique**	**Final determination technique**	**Injected volume [μL]**	**Linearity range [μg/L]**	**Coefficient of determination R^**2**^**	**Precision of the method**				**References**
							**Recovery [%]**	**CV [%]**	**LOD [μg/L]**	**LOQ [μg/L]**	
MEP, MnBP, MEHP, MEHHP	Urine	SPE after enzymatic hydrolysis (β-glucuronidase)	HPLC-MS/MS	20	10–500	0.9869–0.9928	95.2–100.7	1.8–6.0	0.05–3	0.5–8	Mankidy et al., 2013
MEP, MBzP, MiBP, MnBP, MEHP, 5OH-MEHP, 5*oxo*-MEHP	Urine	SPE after enzymatic hydrolysis (β-glucuronidase)	UPLC-MS/MS	10	1–250	0.9950	97–104	7–12	–	0.1–0.5	Servaes et al., 2013
MEP, MnBP, MBzP, MEHP, 5OH-MEHP, 5*oxo*-MEHP, MOP	Urine	LLE after enzymatic hydrolysis (β-glucuronidase)	HPLC-MS/MS	600	0.4–2,000	0.9900	84.6–106	2.5–8.3	0.25–1.0	0.5–2.0	Koch et al., 2003
MMP, MEP, MnBP, MBzP, MEHP, MOP	Urine	SPE after enzymatic hydrolysis (β-glucuronidase)	UPLC-MS/MS	200	1.0–1,000	0.9950	82.5–118.4	2.2–11.3	0.3–0.5	1.0–1.5	Xu et al., 2016
MEP, MnBP, MEHP, MBzP	Urine	SPE after enzymatic hydrolysis (β-glucuronidase)	HPLC-MS/MS	20	5–2,000	0.9968–0.9993	81.8–125.3	0.07–10.2	0.85–5.33	2.8–17.8	Cheng et al., 2014
MEHP, MEHHP, MEOHP, 5cx-MEPP, 2cx-MMHP	Urine	LLE after enzymatic hydrolysis (β-glucuronidase)	UPLC-MS/MS	5	0.5–100	0.9900	90.2–102.0	0.9–12.0	–	1.2–2.6	Monfort et al., 2012
MEP, MiBP, MnBP, MBzP, MiNP, MEHP, MEOHP, MEHHP	Urine	LLE after enzymatic hydrolysis (β-glucuronidase) + derivatization (BSTFA with 1% of TMCS)	GC-MS	2	0.05–100	0.9923–0.9991	61.6–100.1	2.1–16.3	0.05–0.2	0.1–0.5	Kim et al., 2014
MnBP, MiBP, MBzP, MEHP, MEOHP, MECPP, MCPP	Urine	LLE after enzymatic hydrolysis (β-glucuronidase/ arylsulphatase) + derivatization (MTBSTFA)	GC-MS	1	–	>0.995	86.2–136.2	8.6–31.7	5	–	Bamai et al., 2015
MMP, MEP, MnBP, MEHP	Environmental waters, urine	SPME on-fiber derivatization (diazomethane) after enzymatic hydrolysis (β-glucuronidase)	GC-MS	0 (solventless technique)	0.1–150	0.989–0.995	–	14–16	0.1–4.4	0.3–8.6	Alzaga et al., 2003
MiBP, MOP, MMP, MnBP, MCHP, MEHP, MiNP, MBzP	Urine	HF-LPME after enzymatic hydrolysis (β-glucuronidase) with derivatization (BSTFA)	GC-MS	not given	5–1000	0.9747–0.9961	–	12–20	0.77–23	1.2–39	Moreira Fernandeza and André, 2017
MMP, MEP, MnBP, MEHP	Standard solutions	No extraction and derivatization	GC-MS	2	43.5–50000	0.9817–0.9993	–	5.1–9.1	14.5–24.5	43.5–75	Proposed method

*MMP, monomethyl phthalate; MEP, monoethyl phthalate; MnBP, mono-n-butyl phthalate; MBzP, monobenzyl phthalate; MEHP, mono-(2-ethylhexyl) phthalate; MOP, mono-n-octyl phthalate; MiNP, mono-isononyl phthalate; MEHHP, mono-(2-ethyl-5-hydroxyhexyl) phthalate; MEOHP, mono-(2-ethyl-5-oxohexyl) phthalate; 5cx-MEPP/MECPP, mono-(2-ethyl-5-carboxypentyl) phthalate; 2cx-MMHP, mono-(2-carboxymethylhexyl) phthalate; 5OH-MEHP, 5-Hydroxy-mono-(2-ethylhexyl) phthalate; 5oxo-MEHP, 5-oxo-mono-(2-ethylhexyl) phthalate; MCPP, mono-(3-carboxypropyl) phthalate; MCHP, mono-cyclohexyl phthalate; BSTFA, N,O-bis(trimethylsilyl)-trifluoroacetamide; TMCS, trimethyl chlorosilane; MTBSTFA, N-methyl-N-(tert-butyldimethylsilyl)-trifluoroacetamide; SPE, Solid-Phase Extraction; LLE, Liquid-Liquid Extraction; SPME, Solid-Phase Microextraction; HF-LPME, Hollow Fiber Liquid Phase Microextraction; CV, coefficient of variability; LOD, limit of detection; LOQ, limit of quantification*.

Based on the data showed in Table 3 it can be concluded that the proposed method provides the ability to determine monophthalates at the same concentration levels (μg/L). Moreover, with similar precision. It should be noted, that the developed method does not include an isolation and/or enrichment step and therefore, LODs (14.5–24.5 μg/L) and LOQs (43.5–75 μg/L) values are higher than the literature data. If a sample preparation step had been applied, these values would be much lower; however, our goal was to focus more on investigation and explanation of chromatographic mechanisms. Furthermore, the sample volume injections in LC were higher (in the range of 5–600 μL) than in the proposed method (2 μL) which resulted in lower limit values. The obtained basic validation data meets the requirements for analytical procedures and allow determination of trace amounts of monophthalates on levels detected in biological samples. Proposed approach allows for a reduction of analysis time, minimizing possibilities of sample contamination (with regard to the required sample derivatization process; Sagona et al., 2014), reduction of toxic waste production and solvents consumption in comparison to LC. Moreover, the ion suppression effect is minimized, which can be problematic in triple quad MS techniques. On the other hand, the use of hollow fiber liquid phase microextraction simplifies the derivatization step compared to other preparation protocols (Moreira Fernandeza and André, 2017).

Methods with derivatization have already been known and practiced for years. Moreover, after this process the analytes do not require specific chromatographic separation conditions and do not pose a challenge for analysts. To overcome the limitations of this technique, a new approach in the determination of monophthalates using the GC technique was conceived.

The proposed method is based on the splitless injection mode typically used for trace analytes, where the whole volume of injected sample is initially exposed to high injection port temperature and degradation of analytes may occur^5^. During the experiment it was observed that the lowest temperature of vaporization in injector that reduces degradation of analytes was 190°C (no occurrence of phthalic anhydride and 2-ethylhexanol peaks) which is consistent with the fact that the decomposition reaction is strongly temperature dependent. However, such temperature caused splitting of analyte peaks. To overcome the above mentioned problem, pressure pulse mode was used. Higher-pressure injection (at 170 kPa) allows to obtain a better chromatogram peak pattern but also to reduce the volume of sample vapor which is created during the injection by increasing the pressure. The mentioned conditions for the proposed method allow conducting analysis without derivation stage due to lower thermal stress on analytes.

In addition, elimination of derivatization agents from the analytical protocol, which can cause the deterioration of GC stationary phases, increases the service life of the column and makes it more environmentally friendly. On the other hand, using the column with Rtx®-200 phase as retention gap needs better purification of biological samples and more frequent cutting or replacement of the 'guard column'. Replacement of applied retention gap will prevent retention time changes related to its cutting. Also connections between retention gap and analytical column can pose a potential source of leaks and dead volume^6^^,^^7^.

Comparison of the proposed approach with GC-based methodologies available in the literature proves its advantages and enables to obtain satisfactory results with similar precision and accuracy. For example, Bamai et al. (2015) determined phthalate metabolites: mono-*n*-butyl phthalate (MnBP), MiBP, mono (3-carboxypropyl) phthalate (MCPP), mono-benzyl phthalate (MBzP), MEHP, mono(2-ethyl-5-oxohexyl) phthalate (MEOHP), and mono (2-ethyl-5-carboxypentyl) phthalate (MECPP) in urine samples by using LE-GC-MS. The derivatization processes for each metabolite were conducted using 30 μL of MTBSTFA (N-methyl-N-(*tert*-butyldimethylsilyl)-trifluoroacetamide). After addition of MTBSTFA, the solutions were mixed by vortexing at 70°C for 30 min. MEHHP was not measurable because the derivatization of MEHHP did not work well. It should be emphasized, that the entire proposed method takes as much time as the sole derivatization step in the above mentioned process. For all tested phthalate metabolites, the LOD values were determined as 5 μg/L. In this study, the LODs obtained were in the range of 14.5–24.5 μg/L, at a similar concentration level to Bamai et al. It should be noted that the proposed method does not include any isolation and/or enrichment step. If a sample preparation step had been applied, these values would be much lower.

In another study, Kim et al. (2014) examined mono-ethyl phthalate (MEP), mono-ethylhexyl phthalate (MEHP), mono-isobutyl phthalate (MiBP), mono-*n*-butyl phthalate (MnBP), and mono-benzyl phthalate (MBzP) in urine samples by using LE-GC-MS. Trimethylsilanol (TMS) was used as derivatization reagent. The obtained separation and peak shapes were quite similar to those obtained in this study. Moreover, the calculated LODs values are at the same concentration level as in this research. In conclusion, the method proposed in this paper allows for a reduction of analysis time, minimizing possibilities of sample contamination with regard to the required sample derivatization process, which represents an additional step in the analytical procedure and can be a path for the introduction of contaminants to samples or analytes.

## Conclusions

A sensitive (LOD = 14.5–24.5 μg/L) and precise (CV = 1.4–5.4%) method has been developed to investigate the concentration of four monophthalates (monomethyl phthalate, monoethyl phthalate, mono-*n*-butyl phthalate, mono-(2-ethylhexyl) phthalate), which are metabolic products of commonly applied phthalate diesters. It concerns the final determination step in the course of analytical proceedings. In the case of biological samples or other kind of matrix, an effective sample preparation step is necessary. The proposed solution is well-suited for this purpose and allows for a reduction of analysis time, minimizing possibilities of sample contamination (with regard to the required sample derivatization process), reduction of toxic waste production and solvents consumption in comparison to liquid chromatography, while obtaining results with LOD levels similar (if slightly higher) to more complex and time consuming methods. The proposed protocol fulfills the requirements of the Green Analytical Chemistry philosophy, striving to reduce the environmental impact that analytical methods have, since commonly used derivatizing agents are highly toxic and potentially explosive. In addition, their use implies an additional step in the analytical procedure, which can be a path for the introduction of contaminants to samples or analytes.

Higher-pressure injection allows the elimination of the analytes derivatization stage. Therefore, the analytical procedure is simplified by reducing one stage before the qualitative and quantitative determination as well as reducing the risk of mistakes. Thanks to the proposed method, it was possible to solve the limitation of GC in direct determination of monophthalates, which makes it an appropriate and first choice technique in the determination of thermally labile and polar organic compounds.

## Patents

The solution proposed in this paper is the subject of a patent application in the Polish Patent Office No. [WIPO ST10/C PL 427323].

## Data Availability Statement

The raw data supporting the conclusions of this manuscript will be made available by the authors, without undue reservation, to any qualified researcher.

## Author Contributions

MT and LW: conceptualization. MT and AB: methodology and software. MT, LW, and AB: validation. MT and EO: writing—original draft preparation and visualization. LW: supervision.

### Conflict of Interest

The authors declare that the research was conducted in the absence of any commercial or financial relationships that could be construed as a potential conflict of interest.

## Footnotes

^1^Janssen, H. G., Alkema, G., and Kaal, E. *Sample Introduction in Capillary Gas Chromatography*. GL Science B.V. Available online at: https://www.glsciences.eu/optic/gc-injections-manual.pdf (accessed October 15, 2019).

^2^Grob, K. *Sample Evaporation in Splitless Injection. A Problem?* Kantonales Laboratory. Available online at: https://www.restek.com/Technical-Resources/Technical-Library/Editorial/editorial_A021 (accessed October 15, 2019).

^3^*Vapor Volume Calculations of Common Liquids and Solvents*. Agilent Technologies. Available online at: https://www.agilent.com/cs/library/Support/Documents/F03050.pdf (accessed October 15, 2019).

^4^*How Many Injections Can We Get Out of a GC Capillary Column?* Available online at: http://lcgcindia.com/GC%20and%20GC%20MS%20Column/pdf11.pdf (accessed December 2, 2019).

^5^*Minimizing Decomposition of Components During GC Analysis*. Available online at: https://www.restek.com/pdfs/aoi_general_A022.pdf (accessed December 2, 2019).

^6^*What are the Major Causes of GC Capillary Column Performance Degradation?* (2007) Available online at: https://www.agilent.com/cs/library/support/documents/col%20degrd.pdf (accessed December 2, 2019).

^7^de Zeeuw, J. *Using Guard Columns and Retention Gaps in GC (Part 2)*. Restek Corporation. Available online at: https://www.restek.com/Technical-Resources/Technical-Library/Editorial/editorial_A009 (accessed December 2, 2019).
