# Supplementary material for: Advancement in Determination of Phthalate Metabolites by Gas Chromatography Eliminating Derivatization Step
Source: Front Chem. 2020 Jan 15;7:928. doi: 10.3389/fchem.2019.00928 (PMC6974799; doi:10.3389/fchem.2019.00928)
Supplement: Supplementary file 1 [file Data_Sheet_1.pdf]

Supplementary materials

Advancement in determination of phthalate metabolites by gas chromatography eliminating derivatization step

1.1 Experimental design – properties of target compounds

Due to lack of literature data availability, physicochemical properties of monophthalates (vapor pressure, water solubility, octanol-water distribution coefficient) presented in Table S1 were obtained using the EPI™ suite software [U.S. EPA; Tienpont, 2004]. Theoretically calculated data regarding boiling points of MP were confirmed experimentally with reference to di-phthalates (DP). The obtained results are presented in the Figure S1.

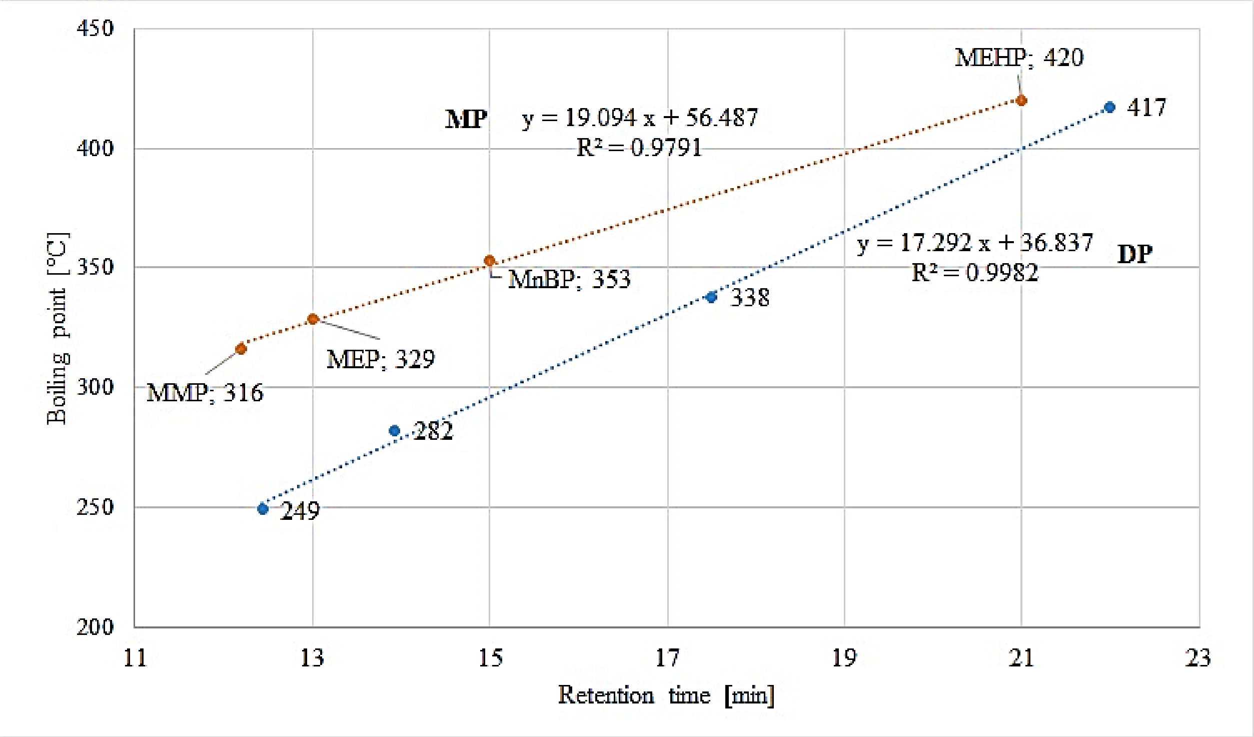

Figure S1. Evaluation of boiling points for studied phthalates

Parent di-phthalate compounds have lower boiling points than their metabolites according to hydrogen bridging of the polar groups. Occurrence of free carboxylic acid group in monophthalates molecules is associated with their higher polar properties and solubility in water compared to their parent compounds. Also, as with di-phthalates, straight chain MPs show higher water solubility and lower octanol-water distribution coefficients than their branched-chain isomers. The solubility of hydrophobic compounds decreases with increasing temperatures [Tienpont, 2004; Thomsen et al., 1999]. As an example, water solubility for DEP varies from 1113 to 741 mg/L when the water

temperature increases by 10 °C. In general, the $K_{o/w}$ parameter (or hydrophobicity) increases proportionately to the molecular weight of monophthalates or the chain length. Additionally, in aqueous matrices, ionized monophthalates are fully solvated. The value of log $K_{o/w}$ for the parent ÷ metabolite pair is higher 1.2 and 1.6 times for DMP ÷ MMP and DnBP ÷ MnBP, respectively. Monophthalates selected for this study exhibit only limited volatility with vapor pressures ranged from $8.15 \cdot 10^{-7}$ to $2.39 \cdot 10^{-4}$ mm Hg at 25 °C. After metabolization of di-phthalates to mono-products, their volatility decreases by one order of magnitude or even more (e.g. for DEP -> MEP vapor pressure changes from $2.54 \cdot 10^{-4}$ to $7.35 \cdot 10^{-5}$ mm Hg) [Tienpont, 2004].

The selected polar monoesters contain a free acid functional group with active hydrogens, which have the tendency to form intermolecular hydrogen bonds. These hydrogen bonds affect the inherent volatility of those compounds, their tendency to interact with materials in the chromatographic system (e.g. GC capillary columns) which causes their irreversible or reversible adsorption and disturb thermal stability [Zaikin and Halket, 2003; Sobolevsky et al., 2003; Lin et al., 2008; Knapp, 1979]. As was mentioned, the derivatization process before GC analysis of trace levels of monoesters is mandatory to block their carboxylic functional group. However, for MEHP classical esterification methods used for fatty acids (e.g. BF_3/KOH/H_2SO_4 and methanol) will lead to further hydrolysis with partial or complete formation of DMP [Tienpont, 2004]. On the other hand, some derivatization reagents also lead to partial hydrolysis of the di-esters and can therefore lead to an overestimation of the results. Most proposed derivatization protocols must be performed in anhydrous conditions which is associated with technical difficulties [Tienpont, 2004; Dirven et al., 1993; Suzuki et al., 2001].

TABLE S1. Basic physicochemical properties of studied monophthalates [U.S. EPA; Tienpont, 2004]

	**Dimethyl phthalate (DMP)**	**Diethyl phthalate (DEP)**	**Di-*n*-butyl phthalate (DnBP)**	**Di-(2-ethylhexyl) phthalate (DEHP)**
**Parent phthalate**	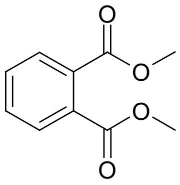	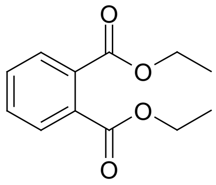	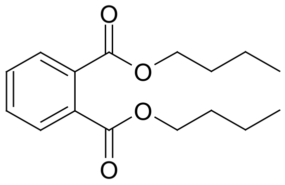	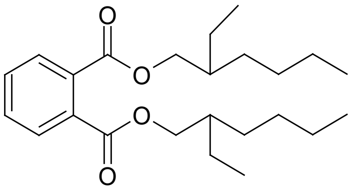
**Molecular weight [g mol^{-1}]**	194.2	222.4	278.4	390.6
	Monomethyl phthalate (MMP)	Monoethyl phthalate (MEP)	Mono-*n*-butyl phthalate (MnBP)	Mono-(2-ethylhexyl) phthalate (MEHP)
**Hydrolytic monoester (primary metabolite of phthalate)**	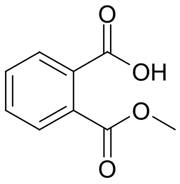	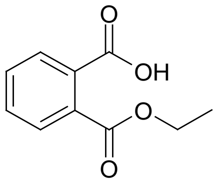	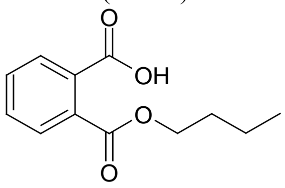	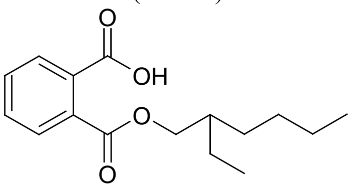
**Molecular formula**	$C_9H_8O_4$	$C_{10}H_{10}O_4$	$C_{12}H_{14}O_4$	$C_{16}H_{22}O_4$
**CAS No.**	4376-18-5	2306-33-4	131-70-4	4376-20-9
		**Physicochemical properties of monoester**		
**Molecular weight [g mol^{-1}]**	180.2	194.2	222.2	278.4
**Boiling point [°C]**	316	329	353	393
**Melting point [°C]**	82-84	n.a.	118	143
**Water solubility [mg L^{-1}] at 25 °C**	5958	1212	125.7	1.492
**log $K_{o/w}$**	1.13	1.86	2.84	4.73
**log P**	1.37	1.77-1.99 (1.66)	2.48-2.96 (2.72)	4.36-4.66 (4.66)
**Vp [mm Hg] at 25 °C**	$2.39 \cdot 10^{-4}$	$7.35 \cdot 10^{-5}$	$3.87 \cdot 10^{-5}$	$8.15 \cdot 10^{-5}$

Legend: log $K_{o/w}$ - octanol-water distribution coefficient, log P - partition coefficient, Vp - vapor pressure, n.a. – not available

1.2 The splitless injection process

According to literature regarding GC theory[1,2] [Grob and Barry, 2004; Biedermann, 2014; Grob Jr et al., 1978; Grob Jr and Grob, 1981], the splitless injection process undergoes the following steps:

- initial GC oven temperature is set approximately 10-20 °C below the boiling point of the sample solvent (in this study: 50 °C as the initial oven temp. and boiling point of methanol is 65 °C),
- split vent line is turned off, the flow rate of carrier gas through injector is relatively slow and almost similar to flow rate through the chromatographic column (in this study: 2.8 mL/min instead of classical conditions of 1 mL/min),
- sample (solvent with analytes) is introduced into a heated injection port as a liquid phase, nebulizes through rapid vaporization of solvent (which results in a local decrease of temperature) and monophthalates present in the aerosol phase,
- the vaporized sample is transferred to the head of the column, where temperature is low enough to recondenseate the solvent wherein analytes re-dissolve in the form of a narrow band (in the presented experiment the sample was trapped at 50 °C). Additionally, the higher pressure applied in the injector compared to the column causes higher transfer rate of vaporized sample to the head of the column (suction effect),
- the GC oven temperature program is set for a rapid increase (10 to 30 °C/min, in this study: 12 °C/min) to allow proper analytes separation.

1.3 Literature

Biedermann, M. (2014). "Inlet Systems and Sample Introduction." in Practical Gas Chromatography, eds. K. Dettmer-Wilde, W. Engewald (Berlin: Springer), 161–203.

Dirven, H.A., van den Broek, P.H., and Jongeneelen, F.J. (1993). Determination of four metabolites of the plasticizer di (2-ethylhexyl) phthalate in human urine samples. Int. Arch. Occup. Environ. Health 64, 555–560.

Grob Jr, K., Grob, G., and Grob, K. (1978). Comprehensive, standardized quality test for glass capillary columns. J. Chromatogr. A 156, 1–20. doi: 10.1016/S0021-9673(00)83120-9

Grob Jr, K., and Grob, K. (1981). Testing capillary gas chromatographic columns. J Chromatogr. A 291, 13–20. doi: 10.1016/S0021-9673(00)80568-3

Grob, R.L., and Barry, E.F. (2004). Modern practice of gas chromatography, 4th ed.; New Jersey: John Wiley & Sons.

---

[1] Technical guide: Operating hints for using split/splitless injectors, Lit. Cat.# 59880A. Restek Corporation. 2002. https://www.restek.com/pdfs/59880A.pdf. (Accessed October 15, 2019).

[2] Chromedia – Gas Chromatography, Injection techniques and principles. The retention gap. 2019. https://chem.libretexts.org/Bookshelves/Analytical_Chemistry/Supplemental_Modules_(Analytical_Chemistry)/Chromedia/01Gas_Chromotography_(GC)/Gas_Chromotography%3A_In_Practice/02Gas_Chromatography%3A_Injection_techniques_and_principles/11The_retention_gap (Accessed October 15, 2019).

Knapp, D.R. (1979). Handbook of analytical derivatization reactions. New York: Wiley- Interscience.

Lin, D.L., Wang, S.M., Wu, C.H., Chen, B.G., and Liu, R.H. (2008). Chemical derivatization for the analysis of drugs by GC-MS - a conceptual review. J Food Drug Anal. 16, 1–10.

Sobolevsky, G., Revelsky, A.I., Miller, B., Oriedo, V., Chernetsova, E.S., and Revelsky, I.A. (2003). Comparison of silylation and esterification/acylation procedures in GC-MS analysis of amino acids. J. Sep. Sci. 26, 1474–1478. doi: 10.1002/jssc.200301492

Suzuki, T., Yaguchi, K., Suzuki, S., and Suga, T. (2001). Monitoring of phthalic acid monoesters in river water by solid-phase extraction and GC-MS determination. Environ. Sci. Technol. 35, 3757–3763. doi: 10.1021/es001860i

Thomsen, M., Rasmussen, A.G., and Carlsen, L. (1999). SAR/QSAR approaches to solubility, partitioning and sorption of phthalates. Chemosphere 38, 2613–2624. doi: 10.1016/S0045-6535(98)00469-X

Tienpont, B. (2004) Determination of phthalates in environmental, food and biomatrices - an analytical challenge. [dissertation]. Ghent University.

U.S. EPA, Estimation Programs Interface Suite™ for Microsoft® Windows, v 3.10. United States Environmental Protection Agency. http://www.epa.gov. (Accessed October 15, 2019).

Zaikin, V.G., and Halket, J.M. Derivatization in mass spectrometry-2. Acylation. (2003). Eur. J. Mass Spectrom. 9, 421–434. doi: 10.1255/ejms.576
